# Effect of Printing Layer Thickness and Postprinting Conditions on the Flexural Strength and Hardness of a 3D-Printed Resin

**DOI:** 10.1155/2022/8353137

**Published:** 2022-02-21

**Authors:** Abdullah A. Alshamrani, Raju Raju, Ayman Ellakwa

**Affiliations:** ^1^Oral Rehabilitation & Dental Biomaterial and Bioengineering, Sydney Dental School, Faculty of Medicine and Health, The University of Sydney, Australia; ^2^Department of Dental Health, College of Applied Medical Sciences, King Saud University, Riyadh, Saudi Arabia; ^3^School of Mechanical and Manufacturing Engineering, UNSW, Sydney, Australia

## Abstract

**Background:**

Recently, dentists can utilize three-dimensional printing technology in fabricating dental restoration. However, to date, there is a lack of evidence regarding the effect of printing layer thicknesses and postprinting on the mechanical properties of the 3D-printed temporary restorations with the additive manufacturing technique. So, this study evaluated the mechanical properties of a 3D-printed dental resin material with different printing layer thicknesses and postprinting methods.

**Methods:**

210 specimens of a temporary crown material (A2 EVERES TEMPORARY, SISMA, Italy) were 3D-printed with different printing layer thicknesses (25, 50, and 100 *μ*m). Then, specimens were 3D-printed using DLP technology (EVERES ZERO, DLP 3D printer, SISMA, Italy) which received seven different treatment conditions after printing: water storage for 24 h or 1 month, light curing or heat curing for 5 or 15 minutes, and control. Flexural properties were evaluated using a three-point bending test on a universal testing machine (ISO standard 4049). The Vickers hardness test was used to evaluate the microhardness of the material system. The degree of conversion was measured using an FT-IR ATR spectrophotometer. Statistical analysis was performed using two-way analysis of variance (ANOVA) and Tukey's honestly significant difference (HSD) test (*p* ≤ 0.05).

**Results:**

The 100 *μ*m printing layer thickness had the highest flexural strength among the other thickness groups. As a combined effect printing thickness and postprinting conditions, the 100 *μ*m with the dry storage group has the highest flexural strength among the tested groups (94.60 MPa). Thus, the group with 100 *μ*m thickness that was heat cured for 5 minutes (HC 5 min 100 *μ*m) has the highest VHN value (VHN = 17.95). Also, the highest mean DC% was reported by 50 *μ*m layer thickness (42.84%).

**Conclusions:**

The thickness of the 100 *μ*m printing layer had the highest flexural strength compared to the 25 *μ*m and 50 *μ*m groups. Also, the postprinting treatment conditions influenced the flexural strength and hardness of the 3D-printed resin material.

## 1. Introduction

Three-dimensional (3D) printing has advanced rapidly in recent years, expanding its accuracy and reliability and making it highly attractive to the medical field. 3D printing has paved the way for new applications in various areas of health care, including medicine, dentistry, orthopedics, engineered tissue models, and medical devices [1–3]. This technology enables the rapid conversion of digital 3D models into physical objects by first producing a digital file in STL (standard triangulation language) format and then 3D printing by joining, bonding, or polymerizing small volume elements [4].

The most popular 3D technologies in the dental field are stereolithography (SLA), digital light projection (DLP), fused deposition modeling (FDM), powder bed fusion (PBF), laser powder forming, and inkjet printing [5, 6]. The main differences between these techniques are the materials used and the way the layers are built to create the 3D object. The two main 3D printing technologies used in dental applications are SLA and DLP. First used for 3D systems in 1986, SLA is a photopolymerization process that builds up solid parts in multilayers on a model-building platform using a photosensitive liquid resin bath and an ultraviolet (UV) light or laser to solidify the material. The layers built by this method are cured and bonded to make a solid object. While the process and system of DLP 3D printing are similar, the photopolymer resin is cured with a digital light projector instead of a laser [7]. DLP 3D printing has an advantage over standard SLA as it enables a faster fabrication of the printed layers by printing and curing a single layer across the total build plate in mere seconds. Another advantage of DLP over SLA, and over other 3D printing systems, is that it consumes less material, thus reducing the cost of production. DLP printing is currently being utilized in the dental industry to create models from digital impressions, such as surgical guides, castable restorations, splints, and even temporary crowns (TC). Due to the speed and accuracy offered by DLP printing, its use is likely to grow within dentistry.

Polymer-based materials are widely used to produce dental crowns using additive technology. However, studies evaluating the use of 3D-printed materials in dentistry in terms of their surface and mechanical properties, including flexural strength, surface roughness, hardness, and esthetics, remain limited. Therefore, the manufacturing process and the strength and polymerization ratio are areas that require further research.

Various parameters can enhance the reliability of 3D-printed materials for use by clinicians and dental technicians, including accuracy, strength, printing speed, and layer thickness [8–10], and curing methods [11–13]. Dental structures and restorative materials are vulnerable to the surrounding environment, and the oral environment is especially challenging due to chemical and thermal variations combined with humidity, which can influence the material properties [14–16]. For example, studies have indicated that the mechanical properties of composite-based resins change due to water storage [17–19]. It was found that the water storage had a negative effect on the flexural properties of a 3D-printed occlusal device material [9]. Furthermore, previous studies have found that artificial aging methods and the polymerization procedure can have adverse effects on the mechanical properties, including fracture resistance and flexural strength, of dental restorations [20, 21]. Flexural strength of a resin-based material with respect to the 3D printing direction has been evaluated in previous studies which they found a positive correlation between the printing directions [9].

Thus, postprocessing treatment such as heat or light curing in some cases is needed for acrylic-based resin in order to cross-link unreacted monomers in order to complete the polymerization process after printing, which improves the final mechanical properties [22, 23]. The amount of polymerization is quantified as the degree of conversion (DC). Therefore, the mechanical properties and biocompatibility are significantly improved with higher DC [24, 25]. This parameter would be more critical for 3D-printed dentures, temporary crowns, and splints that are in contact with soft and hard tissues for much longer times. Previous studies found that using different postcuring equipment resulted in considerable variations in the final properties of the printed devices [20, 26, 27].

Therefore, further research is required into the manufacturing process and postprinting conditions and its effect on mechanical properties of 3D-printed dental materials. Understanding how the mechanical properties of the printed materials are affected by different parameters can help improve the quality of dental restoration and its performance at daily practice.

The selection of a 3D printing material in the dental field depends on the intended application of the end product. For instance, having good mechanical properties and prolonged biodegradation rates is required for dental restoration due to the occlusal forces during the chewing process. Material integration with oral tissue is also one of the factors leading to the success of a dental restoration [28]. Given the high success rate and increasing longevity of dental restoration, especially when fabricated using 3D printing, hence, this study is aimed at evaluating the effect of both printing thickness and postprinting treatment conditions, including water storage, light curing, and heat curing, on the mechanical properties, in terms of the flexural strength and hardness, of 3D-printed temporary crown (TC) materials. The null hypotheses tested were that neither (i) the postprinting method (water storage, light curing, and heat treatment) nor (ii) the printing thickness will significantly change the flexural strength, microhardness. Also, (iii) the postprinting treatment conditions and printing thickness will not significantly change the degree of conversion of a 3D-printed TC resin composite.

## 2. Materials and Methods

**Figure 1:**
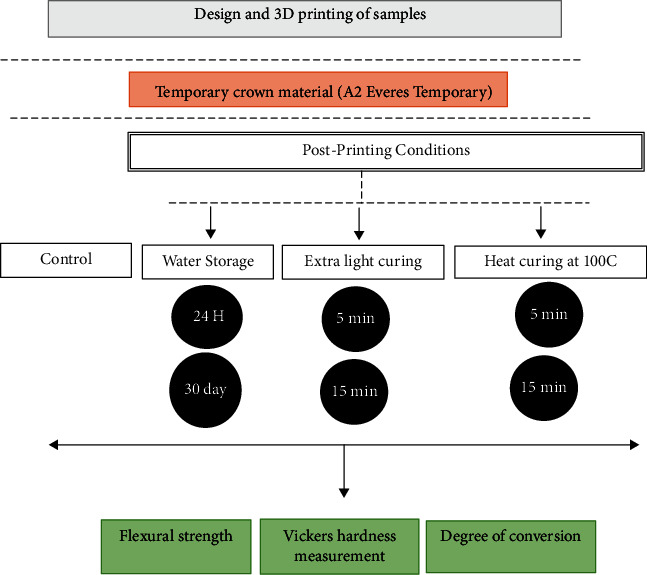
Flowchart of the overall experimental process of this study, showing the materials used, treatment conditions (water storage, extra light curing, and heat curing), and experimental types.

Bar-shaped specimens of light-cured resin materials that used for provisional restorations were 3D-printed (25 × 2 × 2 mm) referring to ISO 4049 [29]. Dental resin (A2 EVERES TEMPORARY, SISMA, Italy) was printed in a DLP 3D printer (EVERES ZERO, DLP 3D printer, SISMA, Italy).  Table 1 presents the composition of the resin material used in the current study. The digital data were exported in STL format. First, the STL file of the sample was imported to DLP 3D printer software to arrange printing sitting. The printing angle was 90° from the print area which was determined based on the results of accuracy evaluations of the thickness, width, and length according to the printing orientation obtained by Tahayeri et al. [8]. The printing support was automatically created at the bottom area of the bar-shaped sample with 0.5 mm point size, 0.85 density, and 3.0 mm height. The printing orientation was determined to be 90 degrees for the build platform. After that, this configuration was duplicated 10 times and 10 identical sample configurations were prepared in the build platform of the DLP printer (EVERES ZERO, DLP 3D printer, SISMA, Italy) to print all samples in the same configuration. This configuration was further saved for 3 different layer thicknesses to print the samples in identical configuration, but by using different layer thicknesses. After the printing process, the specimens were washed out with 90% isopropyl alcohol for 5 minutes according to the manufacturer's instructions and polymerized from all sides for 20 minutes by using ultraviolet light. A total of 210 3D-printed composite resin samples were divided into three main groups with different printing layer thicknesses (25 *μ*m, 50 *μ*m, and 100 *μ*m), at 70 samples each. These three groups were further divided into seven subgroups based on their treatment conditions (water storage, extra light curing, and heat curing). The postprinting conditions were carried out as follows: the water storage group was kept in distilled water in Memmert oven (Memmert, Schwabach, Germany) at a temperature of 37°C for 24 hours and one month. The extra postcuring process was made using light curing unit (Solidilite V, Shofu Dental GmbH, Ratingen, Germany) for 5 and 15 minutes. Finally, the heat curing was carried out using P510 Porcelain Furnace (Ivoclar Vivadent, Schaan, Liechtenstein) at 100°C for 5 and 15 minutes. Finally, the flexural strength (*n* = 10), degree of conversion (*n* = 3), and microhardness (*n* = 5) properties were tested for each experimental group as shown in Figure 1.

### 2.1. Flexural Strength Test

The three-point bending test was performed using a universal testing machine (ISO standard 4049). The specimens were fixed between two supports (20 mm span) and loaded at a crosshead speed of 0.5 mm/min until fracture occurred. The flexural strength was calculated considering the load at fracture and the specimen's dimensions, which were verified using a digital caliper. The fracture load values were converted to flexural strength (*σ*) using the following formula:

(1) $$\begin{array}{c} \sigma =\frac{3FL}{2bd2}, \end{array}$$

where *σ* is the flexural strength, *F* is the load at the fracture point, *L* is the length of the support span, *b* is the width of the specimen, and *d* is the thickness of the specimen.

### 2.2. Vickers Hardness (VH)

The Vickers hardness was measured on the top and bottom surfaces of the specimens using a Struers DuraScan 80 (Cleveland, Ohio, USA) automatic hardness testing system equipped with a Vickers diamond (VHN) with a diamond microindenter, wherein the two diagonals were measured using a load of 500 gmsf (gram force) over 5 s. The surface of each specimen was evaluated five times, and the mean values were calculated for each surface.

### 2.3. Degree of Conversion (DC%)

To measure the degree of conversion, each specimen was placed into the FT-IR ATR spectrophotometer (Bruker, Pty Ltd., Victoria, Australia) using a holder attachment. The Fourier transform infrared spectroscopy (FT-IR) spectra were analyzed using an accessory of reflectance. The absorbance ratio of the material was measured under the following conditions: 32 scans, 4 cm^−1^ resolution, and 300 to 4000 cm^−1^ wavelengths. The percentage of unreacted carbon–carbon double bonds (% C═C) was determined from the ratio of the absorbance intensities of aliphatic C═C (peak at 1714 cm^−1^) to the internal reference of aromatic C═C (peak at 1635 cm^−1^) before and after curing of the specimens. Each specimen was analyzed in triplicate.

The degree of conversion was determined by subtracting the % C═C from 100% according to the following formula:

(2) $$\begin{array}{c} \text{DC}\%=\left(\frac{1–R_{\text{cured}}}{R_{\text{uncured}}}\right)*100, \end{array}$$

where *R*_uncured_ represents the ratio between the intensity of aliphatic C═C (peak at 1714 cm^−1^) and the internal reference of aromatic C═C (peak at 1635 cm^−1^) of the unpolymerized material and *R*_cured_ represents the same ratio after the defined photopolymerization time (*t* = 40 s).

### 2.4. Statistical Analysis

Two-way ANOVA and Tukey's HSD post hoc tests at *α* = 0.05 were used to identify significant differences in terms of flexural strength, hardness, or degree of conversion (dependent variable) according to the main factors, namely, material thickness and treatment conditions (water storage, extra light curing, or heat treatment) (independent variables). All data were subjected to Levene's test of homogeneity of variance (*α* = 0.05).

## 3. Results

### 3.1. Flexural Strength

The mean and standard deviation (SD) of the flexural strength results is presented in Table 2. The two-way ANOVA shows statistically significant differences among the groups (*F*(6,189) = 23.49, *p* < 0.001). Specifically, they indicate a significant influence of water storage, light curing, and heat curing on the flexural strength of the 3D-printed composite resin material. Also, Tukey's HSD post hoc test shows a statistical difference between the test groups. The test result indicates a significant effect of printing thickness on flexural strength (*F*(2,189) = 33.37, *p* < 0.001). The interaction effect of the two factors is also significant (*F*(12,189) = 3.31, *p* < 0.001).

The mean values of the three-point bending test results range from 64.37 to 91.75 MPa for 25 *μ*m thickness, 77.67 to 94.40 MPa for 50 *μ*m, and 66.09 to 94.60 MPa for 100 *μ*m. In general, among the treatment condition groups, the highest flexural strengths were reported for the dry storage group (93.52 MPa). Also, comparing the flexural strength results in terms of the material printing thicknesses, the 50 *μ*m group has the highest value at 86.77 MPa, followed by 79.64 and 74.86 MPa for 100 *μ*m and 25 *μ*m, respectively. As an interaction effect between the main factors (treatment condition and thickness), the 100 *μ*m dry storage group has the highest flexural strength among the tested groups, at 94.60 MPa. In contrast, the lowest flexural strength in terms of treatment conditions is for the one-month water storage group, with a mean value of 72.14 MPa, and in terms of printing thickness, the 25 *μ*m group, with 74.86 MPa.

### 3.2. Vickers Hardness (VH)

Table 2 shows the mean Vickers hardness values for the 3D-printed resin for all tested groups. The results of the two-way ANOVA show that the effects of printing thickness and treatment conditions are statistically significant for microhardness (*p* < 0.05). Regardless of the printing thickness, the heat curing for 5 minutes has increased the mean Vickers hardness values for all groups. In terms of the printing thickness, the results show that the 50 *μ*m group has the highest average Vickers hardness (VHN = 16.68). Also, regarding the interaction effect between the thickness and the treatment conditions, the group with 100 *μ*m thickness that was heat cured for 5 minutes (HC 5 min 100 *μ*m) has the highest VHN value (VHN = 17.95). All additional light and heat curing protocols significantly affected the 100 *μ*m group, which showed an increase in Vickers hardness compared to the control group. For the 50 *μ*m group, the hardness only increased with 15 minutes of heat curing. The 25 *μ*m thickness group only improved when extra light curing was applied for 5 or 15 minutes.

### 3.3. Degree of Conversion (DC%)

**Figure 2:**
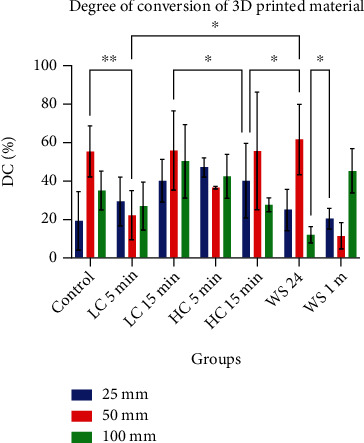
Degree of conversion (DC%) for 25, 50, and 100 *μ*m printing layer thicknesses under different treatment conditions: control (dry storage), light curing for 5 minutes (LC 5 min), light curing for 15 minutes (LC 15 min), heat curing for 5 minutes (HC 5 min), and heat curing for 15 minutes (HC 15 min).

**Figure 3:**
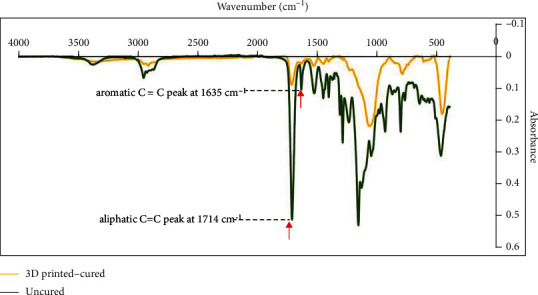
FT-IR spectra showing the absorbance intensities of aliphatic C═C peak at 1714 cm^−1^ to the internal reference of aromatic C═C peak at 1635 cm^−1^ before and after curing of the 3D-printed resin.

The results of the degree of conversion are presented in Figure 2. Also, Figure 3 represents the FT-IR ATR spectra showing the peaks used for calculating the DC%. The two-way ANOVA shows no statistically significant differences between the tested groups. The thickness and postprinting conditions had no effect on the polymerization ratio between the cured and uncured polymers of the 3D-printed material. However, the mean degree of conversion for the 50 *μ*m thickness group (42.84%) is slightly higher than the means of the 25 and 100 *μ*m groups, at 31.63% and 34.16%, respectively.

## 4. Discussion

Additive technology has received significant interest from the dental research community as it can broaden the use of digital applications in dentistry. Dental crowns and bridges are examples of dental devices that can be produced using 3D printing technology. Therefore, understanding the mechanical properties of the dental materials used for fabricating dental crowns is essential for the evaluation of newer 3D printing materials, for verifying the manufacturers' claims, and for comparing them with conventional materials. This will provide a clearer view of which a material is suitable for long-term clinical use. Thus, the aim of this in vitro study was to evaluate the effect of printing layer thickness and different postprinting conditions on the mechanical properties and degree of conversion of a recently developed DLP 3D printing composite resin material. The first and second null hypotheses that the postprinting conditions and water storage will significantly change the flexural strength, microhardness, of a 3D-printed TC resin composite were rejected. However, the third null hypothesis was accepted, demonstrating that the treatment conditions and printing thickness have no effect on the degree of conversion of this 3D-printed temporary material.

Numerous factors can alter the mechanical properties of the printed resin, including build thickness, the degree of polymerization, and the addition of reinforcing materials [30]. Recently, dental research has focused on improving the quality of the printed dental materials, especially dental crowns and bridges, to make them suitable for daily clinical practice; this includes enhancing their durability and biocompatibility [31, 32].

The flexural strength and microhardness of provisional materials are important, particularly when the patient must use a temporary restoration for an extended period until the final restoration has been manufactured [33]. Our flexural strength findings are similar to those of another study comparing the flexural strength and microhardness of a printed resin composite [34] using additive manufacturing, CAD/CAM, and a conventional PMMA material. The mean values of their results were 79.54 MPa for additive manufacturing, 104.20 MPa for CAD/CAM, and 95.58 MPa for the conventional materials [34]. Our flexural strength results are also comparable with other studies of a temporary resin-based material, which found flexural strengths ranging from 60 MPa to 90 MPa [35, 36]. Meanwhile, the lowest flexural strength found in this study was for the one-month water storage group, which had a mean value of 72.14 MPa. On the other side, our findings show that the dry condition produced the highest flexural strength values. This difference between the dry and water storage conditions can be attributed to the water absorption of the temporary dental resin.

A product made by rapid prototyping (RP) technology is influenced by the fabrication technique used during the printing process, which can cause specimen shrinkage during printing and postcuring, or by the minimal thickness of the layers. In addition, data conversion and manipulation in the STL format can also result in changes [37]. Therefore, it can be assumed that the RP resin group has a lower flexural strength than the CAD/CAM group. Our results reveal the low flexural strength of a 3D-printed temporary material compared to the flexural strengths found for CAD/CAM and conventional PMMA resin materials examined in previous studies [9, 34, 38]. Hence, there is a need to improve the mechanical behavior of a 3D-printed material to withstand the mechanical stresses at play during the chewing process. This would increase the durability of a 3D-printed material beyond short-term crowns to permanent and multiunit fixed partial denture (FPD) bridges and crowns. One approach that has been used to this end is the synthesis of nanoparticle fillers in dental resin composites, which has demonstrated further improvements in flexural strength [39], tensile strength, wear resistance [40], and elastic modulus as well as a reduction in the polymerization shrinkage of the material [41].

This in vitro study has evaluated the effect of printing layer thickness on the flexural strength using a three-point bending test. The layers were printed vertically and oriented perpendicular to the load direction. This orientation has been proven at another study to have higher flexural strength comparing to horizontally printed specimens with the layers oriented parallel to load direction [9]. When the layers are parallel to load direction, the junction between the layers is in the path of load direction which leads to the delamination between the layers when the tensile stress is generated during the force application. As a function of printing layer thickness, the three layer thicknesses that are used at the present study were 25, 50, and 100 *μ*m. The higher flexural strength was found at 100 *μ*m. This result was in agreement with the study by Tahayeri et al. [8]. They found that flexural strength was significantly higher for the samples 3D printed with 25 and 100 *μ*m layer thickness, in comparison to the 50 *μ*m layer thickness group.

Another factor crucial to achieving long-term durability is having most of the monomers convert into a polymer during the polymerization process, thereby obtaining an adequate degree of conversion. A low degree of conversion results in inferior mechanical properties, leading to the fast degradation of dental restorations [42]. However, DLP 3D printers use a different process that may influence the material quality and polymerization degree of the final printed restorations. As the printed layers are formed by the photopolymerization of a liquid monomer via an LED source, the different layer thicknesses affect the light penetration into the liquid monomer, which reduces the degree of conversion of the dental resin [43]. Thus, a postcuring process is required to improve the mechanical properties, although the optimal postcuring conditions have not yet been fully determined. In light of this, our study focused on the use of different curing conditions (extra light or heat curing) to ensure the complete polymerization of the 3D resin material as this has been hypothesized to have an effect on the mechanical properties and degree of conversion of the tested material. While the degree of conversion was not significantly changed after exposure to different treatments and curing conditions, this study has shown that the 25 *μ*m printing thickness group had a higher degree of conversion after being heat-cured for 15 minutes than the control group with the same thickness. This may be attributed to a reduced amount of residual monomer due to the extra heat treatment. In general, the increased temperature and extended polymerization time improve the degree of conversion and reduce the release of the monomer [44].

The surface hardness of a material is another crucial mechanical property that can predict the durability of a dental material and its clinical behavior, taking into consideration other parameters such as flexural strength and degree of bond conversion. The surface hardness can be influenced by several other properties, including strength, proportional limit, and wear resistance [45]. The hardness measurements were used in this study to evaluate the various treatment and curing protocols and their effectiveness in terms of polymerization. The results show that the extra light curing and heat curing were effective at increasing the surface hardness at the printing thicknesses of 25 *μ*m and 100 *μ*m. However, the 50 *μ*m thickness was only positively affected when heat cured for 15 minutes. Therefore, it can be highlighted that the type and time of extra curing can positively improve the hardness results of DLP 3D-printed resin, which can be attributed to the depth of curing. Some studies have indicated that the exposure time of curing needs to be considered to achieve a similar depth of cure and ensure optimal performance for resin composite materials, while a correlation has been found between increased hardness and increased degree of conversion [46–48]. Our findings indicate that when the degree of conversion increases in some groups, the hardness also increases, which was shown here for the 50 *μ*m thickness with 15 minutes heat curing. Also, when the 25 *μ*m group was exposed to extra light curing for 15 minutes, the mean Vickers hardness value increased. Therefore, postcuring using UV can improve the mechanical properties of a 3D-printed material by exhausting any residual monomers.

Different 3D printing techniques have been used for various dental applications, and the research has evaluated the dimensional accuracy, flexural strength, and wear resistance of some of these [37, 49, 50]. Hazeveld et al. compared the accuracy of diagnostic casts fabricated using three additive manufacturing technologies (DLP, material jetting (MJ), and PBF), whereby the results showed that DLP is a clinically acceptable method for the fabrication of orthodontic casts [37]. However, another study evaluating the dimensional accuracy of implant replica produced with four different techniques (SLA, DLP, MJ, and conventionally processed stone casts) showed that DLP did not perform significantly better than conventional dental stone for cast duplication [49]. However, using DLP to fabricate interim restorations has promising outcomes in terms of fracture load and flexural strength compared to conventionally processed ones [51]. Overall, different methods can have different outcomes, and a more profound investigation is required to compare these in terms of their mechanical properties and biocompatibility in order to implement them in routine clinical practice.

The findings of this study must be seen in light of some of its limitations. Although there are many additive technologies in use for dental applications, this study evaluated only the mechanical properties and degree of conversion of DLP 3D printing. Therefore, only one technique and one type of a temporary material were used, which is a limitation of this study, and the performance of other 3D printing technologies remains unexplored. It would be beneficial to focus on how other dental materials would perform mechanically using different 3D techniques, including SLA, FDM, PBF, laser powder forming, and inkjet printing [5, 6]. Thus, the second part of this study will focus on a different 3D printing method using various dental materials. Another limitation is the fact that the geometry of the samples used in this work is not similar to that used in clinical settings. Therefore, the crown-shaped samples need to be fabricated in order to simulate the clinical scenario. Other factors can also influence the mechanical properties of a 3D material printed with DLP technology, including printing orientations, water absorption, biocompatibility, long-time survival rate, and color stability of a 3D-printed material. Profound understanding of these aspects will ensure a printed material with the goal of producing crown and bridge materials suitable for better long-term clinical performance and increase the reliability and predictability of dental treatment processes that involve the use of 3D-printed crown and bridge materials [8, 31].

## 5. Conclusion

Within the limitations of the current study and based on the results, the following conclusions can be drawn:

1. The 100 *μ*m 3D printing layer thickness had the highest flexural strength compared to the 25 *μ*m and 50 *μ*m layer thicknesses. However, all of the groups' flexural strength results were higher than the 50 MPa minimum permissible flexural strength of temporary crown materials
2. The 50 *μ*m 3D printing layer thickness group has the highest average Vickers hardness among other groups
3. The 3D printing layer thickness and postprinting treatment conditions had no effect on the degree of conversion ratio of the 3D-printed material

## Figures and Tables

**Table 1:** Material compositions of the EVERES TEMPORARY (SISMA, Italy) 3D-printed resin used in this study.

Material	Compositions	Weight %	Lot#
EVERES TEMPORARY 3D-printed resins	Aliphatic difunctional methacrylate	<50%	276-957-5
	2,2′-Ethylenedioxydiethyl dimethacrylate	<40%	—
	Aliphatic urethane acrylate	<20%	—
	Phosphine oxide	<2.5%	278-355-8

**Table 2:** The means and standard deviations of the Vickers hardness and flexural strength values found for the 3D-printed material.

Postprinting treatment conditions	3D printing thickness					
	20		50		100	
	FS (mean ± SD)^∗^	VHN (mean ± SD)^∗^	FS (mean ± SD)^∗^	VHN (mean ± SD)^∗^	FS (mean ± SD)^∗^	VHN (mean ± SD)^∗^
Dry storage (control)	91.51 (10.05)^a^	13.23 (0.48)^a^	94.40 (9.61)^a^	16.23 (0.22)^a^	91.51 (10.05)^a^	9.31 (0.22)^a^
WS for 24 h	68.38 (9.02)^b^	10.68 (1.67)^a^	88.25 (8.83)^b^	16.63 (0.10)^a^	68.38 (9.02)^b^	12.71 (2.97)^a^
WS for 1 month	64.37 (7.43)^b^	14.86 (1.85)^a^	77.67 (9.13)^bc^	16.38 (0.35)^a^	64.37 (7.43)^b^	13.90 (0.45)^b^
LC 5 min	80.80 (8.72)^ab^	14.33 (0.08)^b^	83.76 (10.44)^c^	16.63 (0.51)^b^	80.80 (8.72)^ab^	12.45 (1.41)^a^
LC 15 min	82.80 (9.09)^ab^	15.21 (0.12)^a^	91.02 (4.80)^ab^	16.20 (1.10)^b^	82.80 (9.09)^ab^	12.93 (0.42)^ab^
HC 5 min	71.32 (4.46)^bc^	13.46 (0.34)^c^	83.47 (12.08)^bd^	17.06 (0.30)^c^	71.32 (4.46)^bc^	17.95 (1.09)^c^
HC 15 min	64.83 (9.07)^bd^	12.41 (0.35)^a^	88.86 (8.03)^bd^	17.66 (0.81)^d^	64.83 (9.07)^bd^	14.88 (0.31)^ad^

^∗^FS: flexural strength; VHN: Vickers hardness. Same superscripted lowercase letters indicate groups not statistically significantly different when compared by Tukey's multiple comparison post hoc analysis (*p* > 0.05).

## Data Availability

The datasets used and/or analyzed during the current study are available from the corresponding author on reasonable request.
